# Multigenic nature of the mouse *pulmonary adenoma progression 1* locus

**DOI:** 10.1186/1471-2164-14-152

**Published:** 2013-03-06

**Authors:** Alice Dassano, Sara Noci, Federica Galbiati, Francesca Colombo, Gaia Trincucci, Angela Pettinicchio, Tommaso A Dragani, Giacomo Manenti

**Affiliations:** 1 Department of Predictive and Preventive Medicine, Fondazione IRCCS, Istituto Nazionale dei Tumori, Via Amadeo 42, Milan, 20133, Italy

**Keywords:** Animal models, CXB recombinant inbred, Disease models, Genome-wide association study, Lung tumors, SNPs, Tumor multiplicity

## Abstract

**Background:**

In an intercross between the SWR/J and BALB/c mouse strains, the pulmonary adenoma progression 1 (*Papg1*) locus on chromosome 4 modulates lung tumor size, one of several measures of lung tumor progression. This locus has not been fully characterized and defined in its extent and genetic content. Fine mapping of this and other loci affecting lung tumor phenotype is possible using recombinant inbred strains.

**Results:**

A population of 376 mice, obtained by crossing mice of the SWR/J strain with CXBN recombinant inbred mice, was treated with a single dose of urethane and assayed for multiplicity of large lung tumors (N2lung). A genome-wide analysis comparing N2lung with 6364 autosomal SNPs revealed multiple peaks of association. The *Papg1* locus had two peaks, at rs3654162 (70.574 Mb, -logP=2.8) and rs6209043 (86.606 Mb, -logP=2.7), joined by an interval of weaker statistical association; these data confirm the presence of *Papg1* on chromosome 4 and reduce the mapping region to two stretches of ~6.8 and ~4.2 Mb, in the proximal and distal peaks, respectively. The distal peak included *Cdkn2a*, a gene already proposed as being involved in *Papg1* function. Other loci possibly modulating N2lung were detected on chromosomes 5, 8, 9, 11, 15, and 19, but analysis for linkage disequilibrium of these putative loci with *Papg1* locus suggested that only those on chromosomes 11 and 15 were true positives.

**Conclusions:**

These findings suggest that *Papg1* consists, most likely, of two distinct, nearby loci, and point to putative additional loci on chromosomes 11 and 15 modulating lung tumor size. Within *Papg1*, *Cdkn2a* appears to be a strong candidate gene while additional *Papg1* genes await to be identified. Greater knowledge of the genetic and biochemical mechanisms underlying the germ-line modulation of lung tumor size in mice is relevant to other species, including humans, in that it may help identify new therapeutic targets in the fight against tumor progression.

## Background

Quantitative trait loci (QTLs) modulating lung tumor size have been mapped in mouse models [1,2]. Lung tumor size, a marker of lung tumor progression in mice, can be expressed as several quantitative traits, including mean tumor volume, total tumor volume or tumor multiplicity (number of tumors larger than a given diameter). One locus specifically modulating mean lung tumor volume in mice, called pulmonary adenoma progression 1 (*Papg1*), has been mapped to a wide region of chromosome 4 included between *D4Mit81* (80.347 Mb) and *D4Mit203* (129.249 Mb), using an intercross between the BALB/c and SWR/J strains [2]. In that study, we observed that animals receiving the BALB/c-derived allele had a ~10-fold greater mean lung tumor volume than those receiving the SWR/J-derived allele [2].

The fine mapping of loci affecting a phenotype may be achieved using recombinant inbred (RI) strains that are generated by crossing two known inbred strains and then inbreeding their descendants. Several RI strains have been developed as mouse models with the aim of mapping different quantitative phenotypes, including lung tumor susceptibility [3,4]. Such an approach is advantageous, since only a single cross is needed and since RI strains may undergo more recombination in the critical QTL interval than a standard intercross population does. The availability of RI strains between BALB/c and SWR/J mice would facilitate the characterization of the *Papg1* locus. In absence of such strains, we considered the series of 13 RI strains between BALB/c and C57BL/6 mice, called CXB. C57BL/6 mice carry a null allele at the *Papg1* locus, as evidenced by the fact that this locus was not detected in crosses of C57BL/6 mice with the genetically susceptible A/J strain but it was instead detected in crosses of A/J with BALB/c mice [5,6] and in crosses between SWR/J and BALB/c mice [2]. Therefore, CXB mice strains represent a suitable model to fine map phenotypic effects associated with BALB/c-derived alleles, especially at the *Papg1* locus.

Herein, we carried out a genome-wide association study on lung tumor susceptibility in a population of CXB mice crossed with the SWR/J strain to further characterize the *Papg1* locus. Our experimental design was similar to the generation of RI intercrosses, which are F1 hybrids between pairs of parental RI lines [7]. In our study, therefore, the mice generated from crossing a mouse from a given CXB line with an SWR/J mouse were isogenic and had a heterozygous genome structure. Our F1 population maintained the same genetic resolution capability of the 13 CXB RI lines: no additional recombinations were introduced by crossing with the SWR/J strain, since both CXB and SWR/J mice are inbred and, therefore, homozygous at all genetic loci. However, introduction of the SWR/J allele at any genetic locus and the expansion of each CXB line allowed us to test the effects of the BALB/c-derived alleles on lung tumor size in the permissive SWR/J genetic background.

Since *Papg1* is the only locus known to specifically modulate lung tumor size in mice, the definition of genes in this locus may help identify new targets of therapy against lung tumor progression. The first steps toward the identification and characterization of such candidate genes are confirming the mapping in independent experiments and reducing the size of the mapping region. To this aim, we carried out a genetic association study in (SWR/J x CXB)F1 mice.

## Results

### Lung tumor phenotypes in CXB RI crossed with SWR/J mice

**Figure 1:**
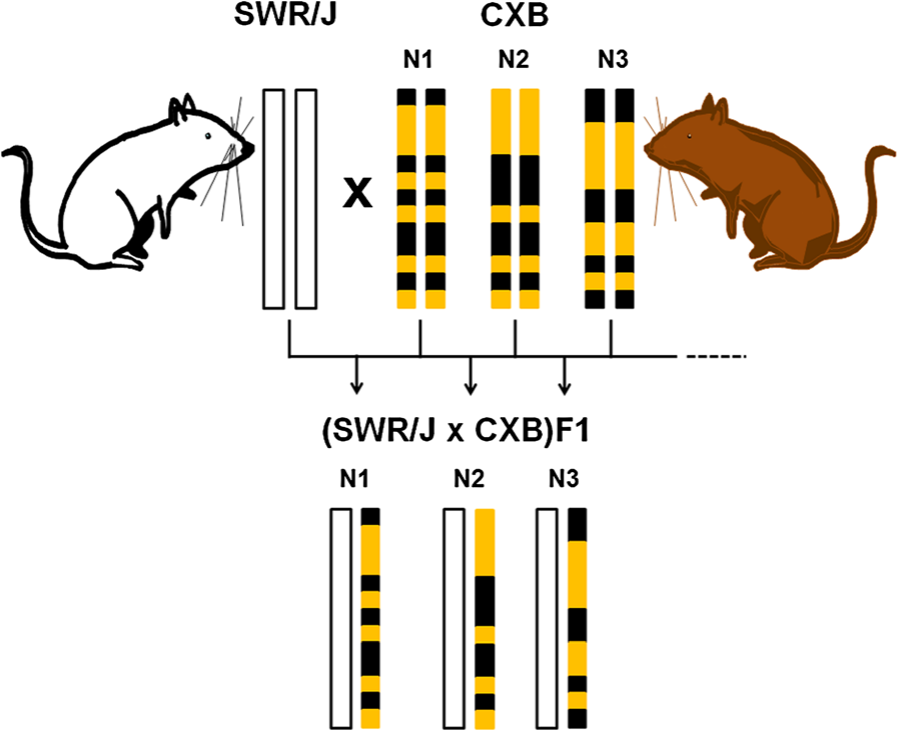
**SWR/J female mice were crossed to male mice belonging to one of the 13 CXB RI strains to obtain a population of 376 male and female (SWR/J x CXBN)F1 mice.** For every locus, each F1 mouse received one copy of the SWR/J allele (white bars) and one copy of the CXBN allele (orange and black bars). Therefore, heterozygosity resulted at every genetic locus, with one allele derived from the SWR/J strain (white bar) and the other derived from either the BALB/c strain (orange band) or the C57BL/6 strain (black band).

SWR/J female mice and CXBN (where N is the number of the RI strain, from 1 to 13) male mice were crossed to generate a population of 376 (SWR/J x CXBN)F1 mice (Figure 1). In these crosses, for each locus, one allele of a CXBN mouse line was replaced with a SWR/J-derived allele. Thus mice belonging to each of the 13 SWR/J x CXBN crosses were genetically identical and heterozygous at all genetic loci, as they consisted of F1 hybrids between inbred strains. At 4 weeks of age, these animals were treated with a single intraperitoneal injection of urethane to induce lung tumors. The experiment was terminated at 40 weeks, when lung tumors were assessed for number and size.

**Figure 2:**
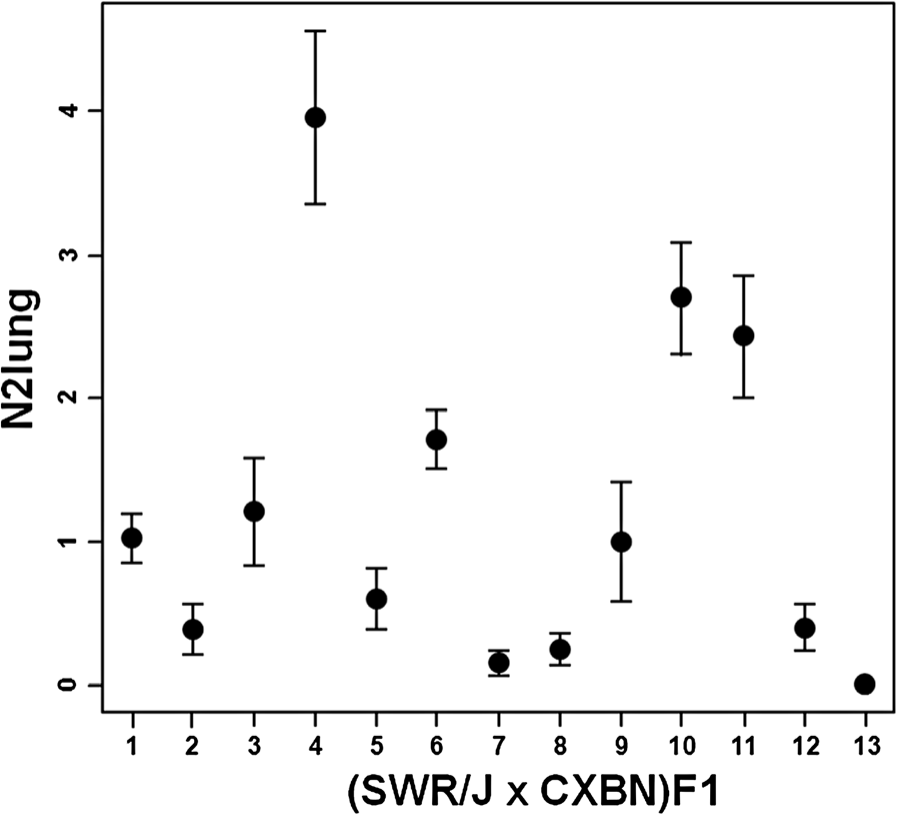
**Multiplicity of lung tumors larger than 2 mm in diameter (N2lung) in (SWR/J x CXBN)F1 mice treated with a single dose of urethane.** For each of the 13 F1 groups derived from various CXBN lines (x-axis), dots represent mean N2lung values and bars represent S.E. of the mean. Number of mice per F1 group ranged from 20 to 35.

In the population of 376 (SWR/J x CXBN)F1 mice treated with urethane, the mean lung tumor volume (Vlung) ranged from 0 to 77.6 mm^3^ in individual animals, giving a population mean of 3.2 mm^3^ (SE=0.4 mm^3^). The number of lung tumors ≥2 mm in diameter (N2lung) ranged from 0 to 12 per animal (mean±SE for the whole population, 1.3±0.1). Overall, 180 of 376 mice (48%) developed one or more lung tumors with diameter ≥2 mm. The 13 different lines showed marked variability in their N2lung phenotype, pointing to the presence of genetic factors modulating this phenotype (Figure 2).

Analysis of sex differences in lung tumorigenesis among the different CXBN crosses with the SWR/J mice showed higher N2lung values (*P*<0.05, Welch’s test) in males than in females in 7 crosses. Therefore, the association analyses between phenotypes and genotypes were adjusted for sex.

### Genome-wide association analysis

A significant correlation was found between Vlung and N2lung (rho=0.85, *P*<2.2 × 10^-16^, Spearman's rank correlation). The high value of the correlation coefficient suggested that the two measures detected the same phenotype. However, N2lung specifically indicates the number of large tumors, whereas Vlung also contains information on small tumors. Therefore, N2lung was judged to be the phenotype more closely related to lung tumor growth and was used in the genome-wide association analysis.

Values of N2lung were square-root-transformed to approximate a normal distribution. Information on 6364 informative autosomal SNPs (815 non-redundant SNPs) in the CXBN lines was obtained from the Wellcome Trust Centre for Human Genetics. The qfam procedure (family-based association tests for quantitative traits) implemented in PLINK 1.05 was used to detect statistically significant associations after adjusting by sex and by performing 10000 permutations per SNP. This procedure, which takes into account family structure, calculated a -logP of 3.7 for the best associated SNP (rs3705264). Had family structure been ignored, i.e. if only a simple linear regression of phenotype on genotype had been performed, the asymptotic *P*-values would have been inflated, e.g. reaching -logP=31.2 for the same marker.

**Figure 3:**
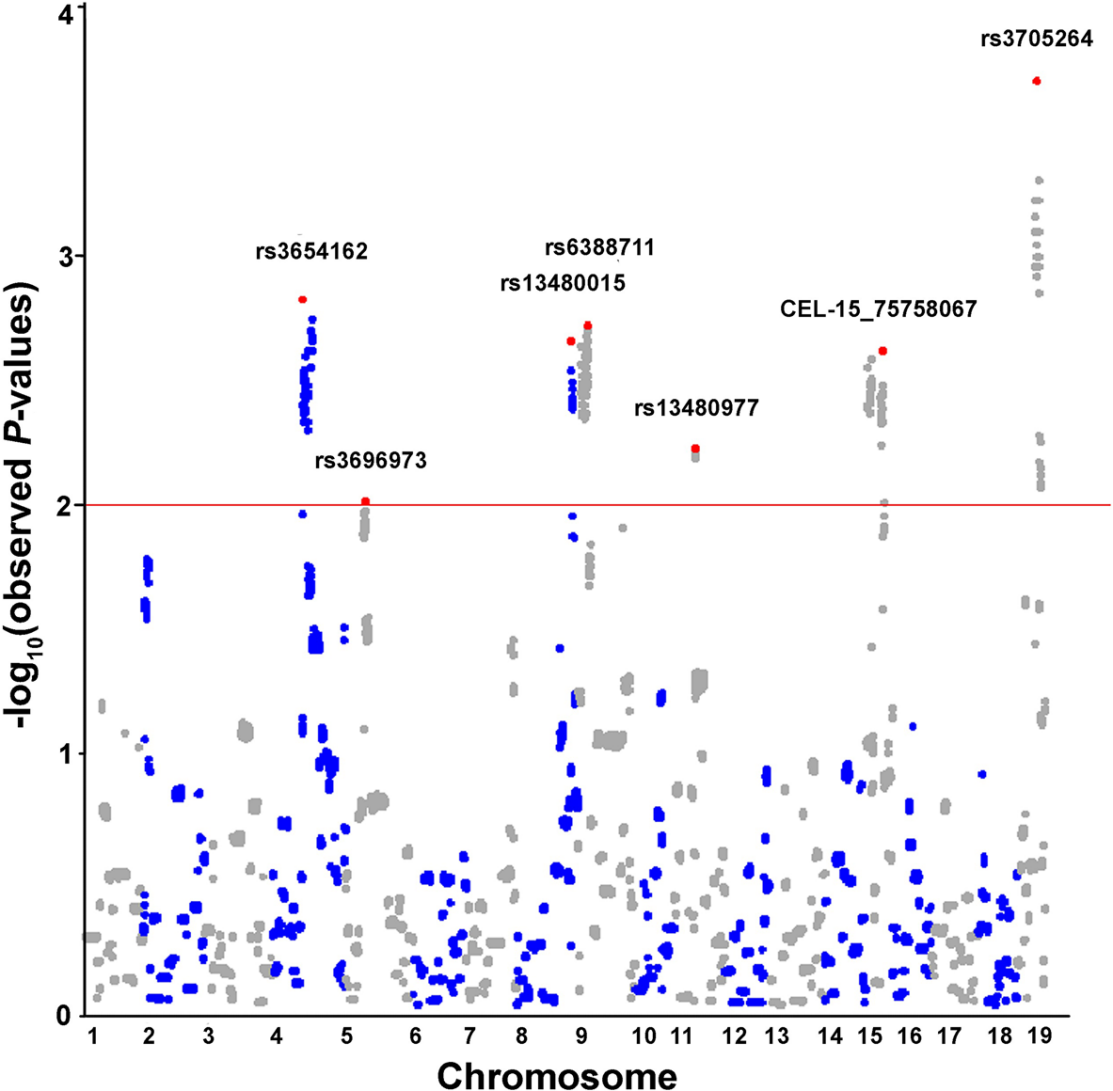
**Manhattan plot of associations between N2lung (square-root-transformed values) and 6364 autosomal SNPs (qfam model, 10000 permutations, PLINK).** The red line indicates the threshold value of P<0.01 for statistical significance. For each chromosome, the SNP with the most significant association above the threshold is marked in red and labeled.

A Manhattan plot of qfam results revealed peaks of association between N2lung and SNPs, above a threshold of empirical *P*-values <0.01 (−logP=2.0; 10000 permutations), on several chromosomal regions (Figure 3). On chromosome 4, rs3654162 at 70.574 Mb (−logP=2.8) and rs6209043 at 89.606 Mb (−logP=2.7) showed the best associations with N2lung phenotype. These data confirm our earlier mapping of the *Papg1* locus on chromosome 4 and narrow the size the mapping region to ~19 Mb. The genome-wide association analysis identified other, new loci putatively modulating N2lung: rs3696973 on chromosome 5 at 55.548 Mb (−log*P*=2.0); rs13480015 on chromosome 8 at 123.159 Mb (−log*P*=2.7); rs6388711 on chromosome 9 at 42.071 Mb (−log*P*=2.7); rs13480977 on chromosome 11 at 39.356 Mb (−log*P*=2.2); CEL-15_75758067 on chromosome 15 at 23.140 Mb (−log*P*=2.6); and rs3705264 on chromosome 19 at 47.045 Mb (−log*P*=3.7).

### Linkage disequilibrium between *Papg1* locus SNPs and other SNPs associated with N2lung

Some of the associations detected between N2lung and SNPs may be false positives because of linkage disequilibrium (LD) between the *Papg1* chromosomal region and unlinked chromosomal regions in the CXBN lines, as has been described for other RI strains [8]. To exclude false-positive loci, we analyzed LD between the two SNPs on chromosome 4 associated with N2lung (rs3654162 and rs6209043) and the best associated SNPs on chromosomes 5, 8, 9, 11, 15, and 19 (Table 1). High values of D’ (>0.5) were observed between the *Papg1*-associated SNP rs3654162 and rs3696973 (chromosome 5), rs13480015 (chromosome 8) and rs3705264 (chromosome 19). For the other *Papg1*-associated SNP, rs6209043, high D’ values were found for rs3696973 (chromosome 5), rs13480015 (chromosome 8), rs6388711 (chromosome 9) and rs3705264 (chromosome 19). In contrast, rs13480977 and CEL-15_75758067, on chromosomes 11 and 15, respectively, showed relatively weak LD with both top SNPs of the *Papg1* locus. These results suggest that the peak associations observed on chromosomes 5, 8, 9, and 19 may be false positives due to LD of the relative regions with the *Papg1* region of chromosome 4. In contrast, the peak associations observed on chromosomes 11 and 15 appear to define new putative lung tumor size modifier loci.

**Table 1:** **Linkage disequilibrium between each of the two top SNPs of the *Papg1 *locus and top SNPs of other putative loci associated with N2lung in (SWR x CXBN)F1 mice**

**Chromosome**	**Position (Mb) **^**a**^	**SNP**	**D' (rs3654162)**	**D' (rs6209043)**
5	55.548	rs3696973	0.76	0.57
8	123.159	rs13480015	1.00	1.00
9	42.071	rs6388711	0.35	1.00
11	39.356	rs13480977	0.07	0.38
15	23.140	CEL-15_75758067	0.46	0.28
19	47.045	rs3705264	0.82	1.00

^a^ Based on NCBI Build 37 assembly (NCBIM37).

### Fine mapping of the *Papg1* locus

**Figure 4:**
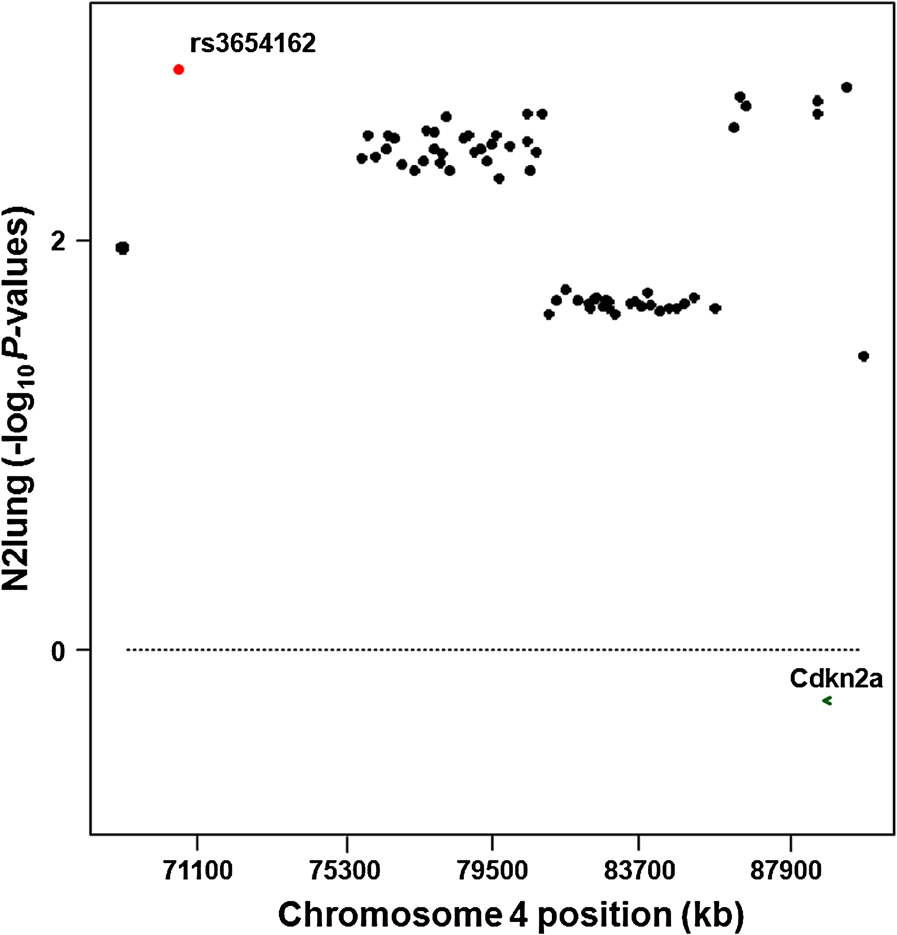
**Results of the association analysis of N2lung (square-root-transformed values) with 62 SNPs on chromosome 4 in the region showing the best statistical associations (P<0.05, from 68938 to 89606 kb) and containing the *Papg1 *locus modulating lung tumor size.** Dots represent values of statistical associations for each SNP, obtained using qfam (PLINK) and reported as minus logarithms of *P* values. The best associated SNP is rs3707373 (−log_10_P=2.8; in red dot). The position of the *Cdkn2a* gene is shown, with an arrow indicating the direction of transcription. Positions of genetic markers on chromosome 4 are based on NCBIM37.

On chromosome 4, rs3654162 had the best association with N2lung phenotype (−logP=2.8). A plot of qfam -logP values across the reduced mapping region of the *Papg1* locus defined by the SNPs rs3654162 and rs6209043 (Figure 4) revealed that, distal to rs3654162 (70.574 Mb), a region of ~5 Mb did not contain informative markers; thus, the two SNPs flanking rs3654162 defined a region of ~6.8 Mb. Then, from 75.775 Mb to 80.950 Mb there was a cluster of 30 SNPs with slightly lower statistical associations (−logP ~2.3-2.6); this was followed by another cluster of 24 SNPs, from 81.112 Mb to 85.878 Mb, with even lower statistical associations (−logP ~1.6-1.8). Finally, the region from 86.396 Mb to 89.606 Mb contained 6 SNPs with -logP values of ~2.6-2.7, just below that of rs3654162; the two SNPs flanking the peak SNP of this region, rs6209043, spanned a ~4.2 Mb long region. These data confirm the existence of the previously mapped proximal region of the *Papg1* locus (80–100 Mb) in the (SWR/J x CXBN)F1 population and indicate that it is composed of two different peaks. However, these data do not confirm, in this population, the remainder of the locus from 100 to 129 Mb.

Looking in greater detail at the genetic content of the more narrowly mapped *Papg1*, the best *Papg1*-associated SNP, rs3654162 at 70.574 Mb, maps to an intergenic region between the *Megf9* gene (multiple EGF-like-domains 9) and the predicted gene *Gm11228*. In the ~6.8 Mb region delimited by the two markers that flank this SNP, namely rs13477759 and rs13477785, there are 61 genes or predicted genes. In the distal associated region delimited by SNPs rs3711477 and rs3682389 and spanning 4.2 Mb, 91 genes or predicted genes map. The best associated SNP in this region, rs6209043, is located in an intergenic region flanked by *Dmrta1* (doublesex and mab-3 related transcription factor like family A1) and the predicted gene *Gm12629*. About 300 kb proximal to rs6209043 maps *Cdkn2a*, a gene previously proposed for candidacy of *Papg1* functions [9].

**Figure 5:**
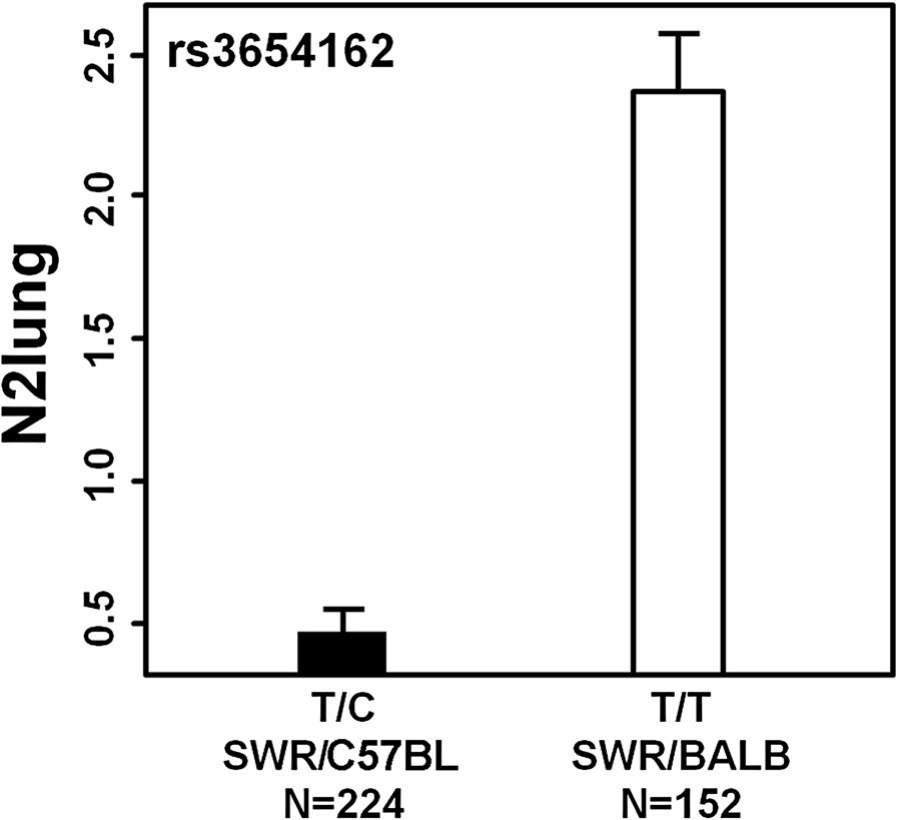
**Effects of the BALB/c- and C57BL/6-derived alleles on N2lung values in (SWR/J x CXBN)F1 mice at rs3654162 (70.574 Mb).** For every gene, each F1 mouse carried one SWR/J (SWR)-derived allele and one allele from either the C57BL/6J strain (C57BL) or the BALB/c/c strain (BALB). The genotype of the genetic marker is reported on the x-axis, together with the strain derivation of the alleles and the number of mice. Values are mean and S.E. of the mean.

Phenotype-by-genotype analysis showed that (SWR/J x CXBN)F1 animals that inherited the BALB/c-derived allele at the proximal best associated SNP (rs3654162) had a ~5-fold higher mean value of N2lung than did animals inheriting the C57BL/6J allele (Figure 5). Similar findings were observed at the distal best associated SNP (rs6209043; -logP=2.7): mice inheriting the BALB/c-derived allele had a ~5-fold higher mean value of N2lung than mice inheriting the C57BL/6J allele. These finding are in agreement with our previous observations showing the association of large lung tumors with the BALB/c-derived allele at the *Papg1* locus [2].

## Discussion

In the present study, 4-week-old (SWR/J x CXBN)F1 intercross mice were treated with urethane to induce lung neoplasia and evaluated at age 40 weeks for lung tumor multiplicity. Using the number of large lung tumors (≥2 mm in diameter; N2lung), we carried out a genome-wide association study on 6364 SNPs to identify loci that modulate lung tumor progression. Two distinct but nearby peaks of association were found on chromosome 4, confirming the mapping of the *Papg1* locus in a new cross and narrowing the size of the mapping region to two stretches of 6.8 and 4.2 Mb. Additional peaks of association were found on chromosomes 5, 8, 9, 11, 15, and 19, but an analysis of LD excluded those on chromosomes 5, 8, 9 and 19 as false positives leaving two possible new loci on chromosomes 11 and 15.

To study lung tumorigenesis, we induced neoplastic lesions by a single injection of urethane. Since this chemical carcinogen has a short half-life (<1 h) [10], tumor initiation began at essentially the same time point in all animals. The two variables of lung tumor formation initially studied, Vlung and N2lung, were highly correlated. However, since N2lung referred to large tumors, we judged it to be more representative of lung tumor progression. This variable is an easy to assess quantitative measure of lung tumor size, which is a surrogate of lung tumor progression phenotype. Indeed, it represents the tendency of an individual mouse to develop a low or high frequency of tumors of large size in a defined time interval. And, although the propensity of individual tumors to progress may be due to somatic alterations [11], differences in mean size of lung tumors among groups of isogenic animals reflects the genetic predisposition of these animals to lung tumor progression.

The fact that two peaks of association were detected on chromosome 4, separated by a valley (~16-18 Mb) of weak statistical association, suggests that *Papg1* is a complex multigenic locus containing at least two and possibly more genes whose different alleles modulate lung tumor progression in mice. The distal peak of association contains, in its distal part, *Cdkn2a*, mapping at 88.920-88.941 Mb. This gene had previously been proposed as a candidate gene for *Papg1* since, in A/J and BALB/c crosses treated with urethane and analyzed for lung tumor multiplicity, it maps in the linkage region of chromosome 4 [6]. This candidacy is therefore confirmed by the present study. However, the QTL curve of the earlier study showed low linkage values in the proximal part of *Papg1* where rs3654162, the SNP with the best association in our study, was mapped (~18 Mb proximal to *Cdkn2a*). These discrepant results for the proximal peak may reflect the different durations of the period after urethane treatment (about 21 weeks vs. 36 weeks in the present study) or the different strains crossed with BALB/c mice (A/J vs. SWR/J).

An additional study proposed *Cdkn2a* as a candidate gene for the *Papg1* locus based on the observation that mice with a heterozygous deficiency for the A/J-derived *Cdkn2a* allele were significantly more susceptible to lung tumor progression than mice with a heterozygous deficiency for the BALB/cJ-derived *Cdkn2a* allele [9]. These data suggested that the A/J-derived allele of *Cdkn2a* is a more potent tumor suppressor than the BALB/cJ-derived allele and pointed to *Cdkn2a* as being responsible for *Papg1* function.

We cannot exclude that additional genes are involved in *Papg1* functions since a large number of genes, predicted genes and small nuclear RNAs map in the *Papg1* locus. The proximal region of this locus, near rs3654162, contains *Megf9* and *Tle1*, which are both known to be involved in tumorigenesis. In particular, TLE1 is a transcriptional repressor protein whose overexpression promotes resistance to anoikis (apoptosis after loss of cell attachment to the extracellular matrix) in human breast carcinoma cells [12]. It was also found to be upregulated in invasive human breast cancer compared to noninvasive carcinoma and normal mammary epithelial tissue [12]. Expression of MEGF9, a transmembrane protein with multiple EGF-like domains, was associated with local aggressiveness in human soft tissue tumors [13]. The distal region of the *Papg1* locus contains, besides *Cdkn2a*, several other genes, mostly belonging to the interferon family (data not shown); it is therefore possible that the function of this locus is due to some gene involved in the antitumoral immune response [14]. Another gene in this region is *Mtap,* known to be a tumor suppressor gene since mice with heterozygous *Mtap* germ-line mutations developed T-cell lymphoma and died prematurely [15]. Additional studies could clarify the roles, if any, of these genes in lung tumor progression.

A further refinement of the map of this locus is needed to reduce the size of the chromosomal region functionally linked with modulation of lung tumor size and, consequently, the number of candidate genes. This refinement may be achieved by marker-assisted backcrossing of the BALB/c-derived allele into the SWR/J genetic background to generate congenic strains, as has already been done for the functional cloning of candidate genes of loci in other mouse crosses [16].

Additional loci associated with N2lung, not previously reported in (BALB/c x SWR/J)F2 or (BALB/c x A/J)F2 intercrosses, were detected on other chromosomes. Loci on chromosomes 5, 8, 9 and 19 were determined to be false positives due to LD with *Papg1,* whereas the loci on chromosomes 11 and 15, not in LD with *Papg1,* may be true additional loci modulating lung tumor size resulting from the introduction of the C57BL/6 genome in the present cross. It should be noted, however, that a lung tumor modifier locus (designed *Pas3*) had been mapped on chromosome 19, in a position slightly proximal to the one herein identified, in an intercross population between A/J and C57BL/6J mice [17]. Further research should determine if chromosome 19 contains another tumor-modifying locus.

## Conclusions

This study shows the complex multigenic nature of *Papg1*, which appears to contain two or more genes modulating lung tumor progression in mice. A strong candidate for this locus is *Cdkn2a,* while the identification of other candidate genes requires additional studies. Moreover, the study provides evidence for putative new loci affecting lung tumor size, thereby adding additional genetic elements to the complex control of lung tumor progression. Our results encourage further research to identify additional candidate *Papg1* genes to understand the mechanisms of germ-line modulation of lung tumor progression in mice. Such knowledge may be of relevance to other species, including humans, in the identification of genes modulating lung tumor progression.

## Methods

### Mice, treatment, and phenotypes

SWR/J female mice and CXBN (N=1 to 13) male mice were purchased from Charles River Italia (Calco, Italy). Male and female mice were crossed in our husbandry unit to generate a population of 376 (SWR/J x CXBN)F1 mice (216 males and 160 females). At 4 weeks of age, these animals were treated with a single intraperitoneal injection of urethane (1000 mg/kg body weight) dissolved in water. The experiment was terminated at 40 weeks, when the animals were killed and their lungs were fixed in formalin.

All animals received humane care according to the criteria outlined in the Guide for the Care and Use of Laboratory Animals of the US National Research Council, 1996. Experiments were approved by C.E.S.A. (Ethical Committee for Animal Experimentation, Fondazione IRCCS Istituto Nazionale dei Tumori, Milan, Italy) and by the Italian Ministry of Health, according to the Italian law DL 116/92, as part of the project entitled: “Identification and characterization of genes modulating susceptibility and resistance to cancer”, 2007–2009.

The number and size of lung tumors were assessed at gross examination by counting and measuring neoplastic lesions in each formalin-fixed lung lobe. Data were reported according to two lung tumor-related phenotypes: mean lung tumor volume (Vlung), calculated assuming that all tumors have a spherical shape, and number of lung tumors with diameter ≥2 mm (N2lung).

### Data downloading and genome-wide association analysis

Information on the genotypes of the CXBN lines was obtained from the Wellcome Trust Centre for Human Genetics (WTCHG) (http://www.well.ox.ac.uk/mouse/INBREDS/). Data were used for all SNPs except for those on chromosome X, since all (SWR/J x CXBN)F1 male mice had inherited the identical chromosome X from the SWR/J female parent, and those with missing genotype data. The SNP genotypes of (SWR/J x CXBN)F1 mice were assigned according to those of their parents. The physical positions of SNPs were based on NCBI Build 37 assembly (NCBIM37). The number of genes mapping in specific chromosomal regions was extracted from the Ensembl web site (http://www.ensembl.org/Mus_musculus/Info/Index), using the BioMart tool.

### Statistical analyses

Correlation was assessed using Spearman's rank correlation coefficient. Analysis of sex differences in lung tumor multiplicity was carried out using Welch’s *t* test. Statistical procedures were carried out using R or the “Rcmdr” library in R [18] and all statistical tests were two-sided.

Associations between SNPs and N2lung were assessed with PLINK software, using qfam, a family-based association test for quantitative traits. This test performs a simple linear regression of phenotype on genotype, and then uses a special permutation procedure to correct for family structure and to calculate empirical P-values [19]. These analyses were adjusted by sex. Linkage disequilibrium analyses between markers were also carried out using PLINK. Manhattan plots were generated using the “gap” library in R.

## Competing interests

The authors have no interest, arrangement, or affiliation that could be perceived as a conflict of interest in the context of this manuscript.

## Authors’ contributions

AD downloaded the SNP data, managed the database, and drafted and finalized the manuscript. AP, FC, FG, GT, and SN were involved in many aspects of the study, including animal crossing, treatment, autopsy, and phenotype analysis. TAD performed the statistical analysis and revised the manuscript. GM supervised the study and revised the manuscript. All authors read and approved the final manuscript.
